# 1-Benz­yloxy-1*H*-benzotriazole

**DOI:** 10.1107/S1600536812028395

**Published:** 2012-06-30

**Authors:** Samuel Robinson Jebas, P. Selvarathy Grace, B. Ravindran Durai Nayagam, Dieter Schollmeyer

**Affiliations:** a Department of Physics, Sethupathy Government Arts College, Ramanathapuram 623 502, Tamilnadu, India; b Department of Chemistry, Popes College, Sawyerpuram 628 251, Tamilnadu, India; c Institut für Organische Chemie, Universität Mainz, Duesbergweg 10–14, 55099 Mainz, Germany

## Abstract

In the title compound, C_13_H_11_N_3_O, the dihedral angle between the benzotriazole ring system [maximum deviation = 0.027 (16) Å] and the benzene ring is 10.28 (9)°. The C—C—O—N bond adopts an *anti* conformation [torsion angle = −177.11 (16)°]. In the crystal, the mol­ecules inter­act *via* weak C—H⋯π inter­actions and aromatic π–π stacking [centroid-to-centroid distance = 3.731 (12) Å].

## Related literature

For a related structure and background to benzotriazoles, see: Selvarathy Grace *et al.* (2012 ▶).
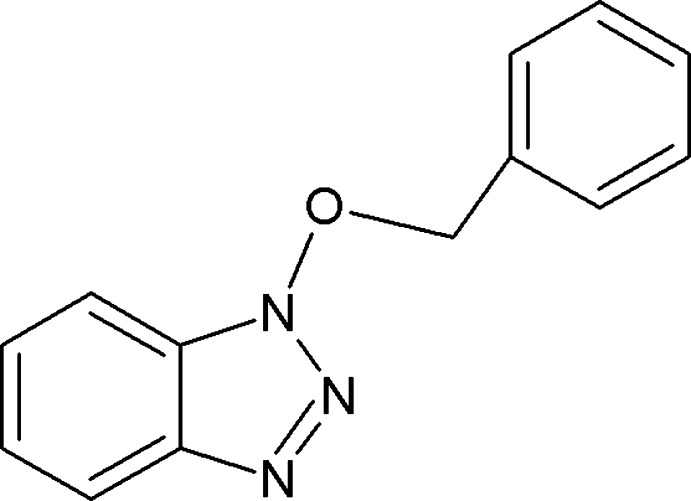


## Experimental

### 

#### Crystal data

C_13_H_11_N_3_O
*M* *_r_* = 225.25
Orthorhombic, 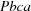

*a* = 11.2417 (5) Å
*b* = 7.8381 (8) Å
*c* = 25.3933 (18) Å
*V* = 2237.5 (3) Å^3^
*Z* = 8
Cu *K*α radiation
μ = 0.72 mm^−1^
*T* = 193 K
0.51 × 0.45 × 0.13 mm

#### Data collection

Enraf–Nonius CAD-4 diffractometer
Absorption correction: ψ scan (*CORINC*; Wiehl & Schollmeyer, 1994 ▶) *T*_min_ = 0.84, *T*_max_ = 0.99
2125 measured reflections
2125 independent reflections
1867 reflections with *I* > 2σ(*I*)
*R*_int_ = 0.000
3 standard reflections every 60 min intensity decay: 3%

#### Refinement

*R*[*F* ^2^ > 2σ(*F* ^2^)] = 0.054
*wR*(*F* ^2^) = 0.163
*S* = 1.12
2125 reflections
155 parameters
H-atom parameters constrained
Δρ_max_ = 0.26 e Å^−3^
Δρ_min_ = −0.23 e Å^−3^

### 

Data collection: *CAD-4 Software* (Enraf–Nonius, 1989 ▶); cell refinement: *CAD-4 Software*; data reduction: *CORINC* (Wiehl & Schollmeyer, 1994 ▶); program(s) used to solve structure: *SHELXS97* (Sheldrick, 2008 ▶); program(s) used to refine structure: *SHELXL97* (Sheldrick, 2008 ▶); molecular graphics: *SHELXTL* (Sheldrick, 2008 ▶); software used to prepare material for publication: *PLATON* (Spek, 2009 ▶).

## Supplementary Material

Crystal structure: contains datablock(s) global, I. DOI: 10.1107/S1600536812028395/hb6865sup1.cif

Structure factors: contains datablock(s) I. DOI: 10.1107/S1600536812028395/hb6865Isup2.hkl

Supplementary material file. DOI: 10.1107/S1600536812028395/hb6865Isup3.cml

Additional supplementary materials:  crystallographic information; 3D view; checkCIF report

## Figures and Tables

**Table 1:** Hydrogen-bond geometry (Å, °) *Cg*1 is the centroid of the C12–C17 benzene ring.

*D*—H⋯*A*	*D*—H	H⋯*A*	*D*⋯*A*	*D*—H⋯*A*
C13—H13⋯*Cg*1^i^	0.95	2.86	3.685 (2)	145
C16—H16⋯*Cg*1^ii^	0.95	2.99	3.691 (3)	132

Symmetry codes: (i) 
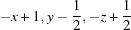
; (ii) 
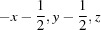
.

**Fig. 1.**
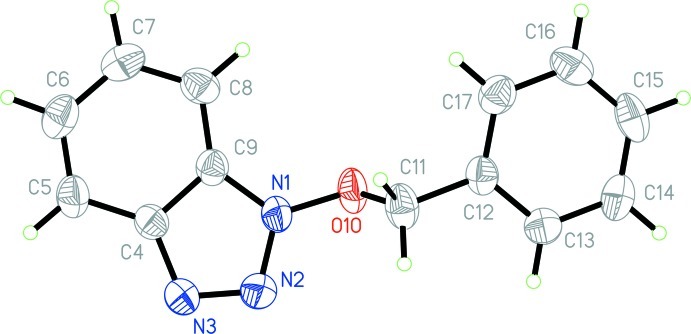
The asymmetric unit of the title compound, showing displacement ellipsoids drawn at the 50% probability level.

**Fig. 2.**
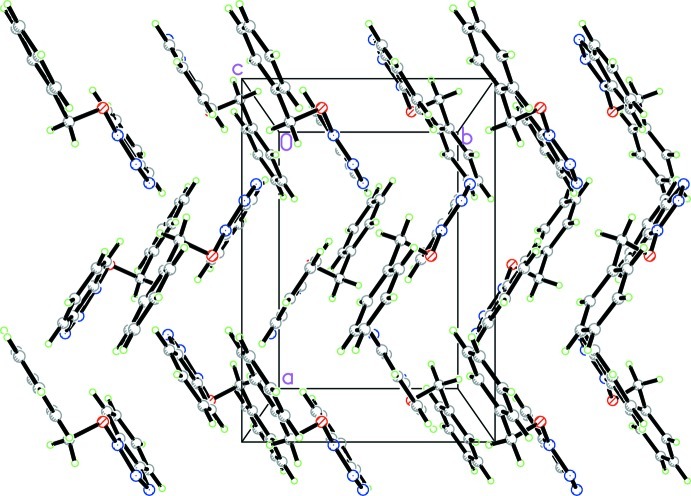
The crystal packing of the title compound, viewed along the c axis.

## supplementary crystallographic information

### Comment

As part of our ongoing studies of benzotriazole derivatives with possible biological activities (Selvarathy Grace *et al.*, 2012) we now report the crystal structure of the title compound (I).

The benzotriazole ring is essentially planar with the maximum deviation from planarity being 0.027 (16) Å for atom N2. The mean plane of the benzotriazole ring (N1–N3/C4–C9 forms a dihedral angle of 10.28 (9)° with the mean plane of the phenyl ring (C12–C17).

The crystal packing features π–π stacking interactions with the centroid-centroid distance of 3.731 (12) Å [symmetry code: 1 - x, -y, 1 - z], together with weak C—H···π interactions. Molecules are stacked along the *b* axis (Fig 2).

### Experimental

A mixture of sodium salt of 1-hydroxy benzotriazole (0.157 g, 1 mmol) and benzyl chloride (0.126 g, 1 mmol) in ethanol and water (10 ml), were heated at 333 K with stirring for 6 h. The mixture was kept aside for slow evaporation. After a week, colourless blocks were obtained.

### Refinement

H atoms were positioned geometrically [C—H = 0.95 (aromatic) or 0.99 Å (methylene)] and refined using a riding model, with *U*_iso_(H) = 1.2*U*_eq_(C).

### Crystal data

**Table:**

C_13_H_11_N_3_O	*F*(000) = 944
*M**_r_* = 225.25	*D*_x_ = 1.337 Mg m^−^^3^
Orthorhombic, *P**b**c**a*	Cu *K*α radiation, λ = 1.54178 Å
Hall symbol: -P 2ac 2ab	Cell parameters from 25 reflections
*a* = 11.2417 (5) Å	θ = 60–70°
*b* = 7.8381 (8) Å	µ = 0.72 mm^−^^1^
*c* = 25.3933 (18) Å	*T* = 193 K
*V* = 2237.5 (3) Å^3^	Block, colourless
*Z* = 8	0.51 × 0.45 × 0.13 mm

### Data collection

**Table:**

Enraf–Nonius CAD-4 diffractometer	1867 reflections with *I* > 2σ(*I*)
Radiation source: rotating anode	*R*_int_ = 0.000
Graphite monochromator	θ_max_ = 70.0°, θ_min_ = 3.5°
ω/2θ scans	*h* = 0→13
Absorption correction: ψ scan (CORINC; Wiehl & Schollmeyer, 1994)	*k* = −9→0
*T*_min_ = 0.84, *T*_max_ = 0.99	*l* = 0→30
2125 measured reflections	3 standard reflections every 60 min
2125 independent reflections	intensity decay: 3%

### Refinement

**Table:**

Refinement on *F*^2^	Secondary atom site location: difference Fourier map
Least-squares matrix: full	Hydrogen site location: inferred from neighbouring sites
*R*[*F*^2^ > 2σ(*F*^2^)] = 0.054	H-atom parameters constrained
*wR*(*F*^2^) = 0.163	*w* = 1/[σ^2^(*F*_o_^2^) + (0.0919*P*)^2^ + 0.968*P*] where *P* = (*F*_o_^2^ + 2*F*_c_^2^)/3
*S* = 1.12	(Δ/σ)_max_ < 0.001
2125 reflections	Δρ_max_ = 0.26 e Å^−^^3^
155 parameters	Δρ_min_ = −0.23 e Å^−^^3^
0 restraints	Extinction correction: *SHELXL97* (Sheldrick, 2008), Fc^*^=kFc[1+0.001xFc^2^λ^3^/sin(2θ)]^-1/4^
Primary atom site location: structure-invariant direct methods	Extinction coefficient: 0.0022 (5)

### Special details

**Table:**

Geometry. All esds (except the esd in the dihedral angle between two l.s. planes) are estimated using the full covariance matrix. The cell esds are taken into account individually in the estimation of esds in distances, angles and torsion angles; correlations between esds in cell parameters are only used when they are defined by crystal symmetry. An approximate (isotropic) treatment of cell esds is used for estimating esds involving l.s. planes.
Refinement. Refinement of F^2^ against ALL reflections The weighted R-factor wR and goodness of fit S are based on F^2^, conventional R-factors R are based on F, with F set to zero for negative F^2^. The threshold expression of F^2^ > 2sigma(F^2^) is used only for calculating R-factors(gt) etc. and is not relevant to the choice of reflections for refinement. R-factors based on F^2^ are statistically about twice as large as those based on F, and R-factors based on ALL data will be even larger.

### Fractional atomic coordinates and isotropic or equivalent isotropic displacement parameters (Å^2^)

**Table:**

	*x*	*y*	*z*	*U*_iso_*/*U*_eq_	
N1	0.59619 (14)	0.1616 (2)	0.41567 (6)	0.0319 (4)	
N2	0.69169 (16)	0.0710 (2)	0.40089 (7)	0.0386 (5)	
N3	0.74822 (17)	0.0253 (3)	0.44366 (6)	0.0390 (5)	
C4	0.68515 (16)	0.0849 (3)	0.48621 (7)	0.0300 (4)	
C5	0.70541 (19)	0.0634 (3)	0.54041 (8)	0.0363 (5)	
H5	0.7724	0.0020	0.5531	0.044*	
C6	0.6242 (2)	0.1348 (3)	0.57406 (8)	0.0395 (5)	
H6	0.6348	0.1216	0.6110	0.047*	
C7	0.5253 (2)	0.2276 (3)	0.55539 (8)	0.0390 (5)	
H7	0.4719	0.2767	0.5802	0.047*	
C8	0.50350 (18)	0.2493 (3)	0.50265 (9)	0.0342 (5)	
H8	0.4366	0.3111	0.4900	0.041*	
C9	0.58618 (17)	0.1744 (2)	0.46878 (7)	0.0285 (4)	
O10	0.51465 (12)	0.21382 (19)	0.37887 (5)	0.0364 (4)	
C11	0.56357 (18)	0.3549 (3)	0.34807 (8)	0.0373 (5)	
H11A	0.6347	0.3171	0.3283	0.045*	
H11B	0.5867	0.4503	0.3715	0.045*	
C12	0.46776 (17)	0.4101 (3)	0.31104 (7)	0.0296 (5)	
C13	0.47315 (18)	0.3682 (3)	0.25810 (8)	0.0352 (5)	
H13	0.5377	0.3018	0.2453	0.042*	
C14	0.3853 (2)	0.4220 (3)	0.22357 (8)	0.0400 (5)	
H14	0.3901	0.3931	0.1873	0.048*	
C15	0.29088 (19)	0.5177 (3)	0.24185 (9)	0.0398 (5)	
H15	0.2310	0.5551	0.2181	0.048*	
C16	0.28349 (19)	0.5589 (3)	0.29457 (9)	0.0403 (5)	
H16	0.2177	0.6229	0.3073	0.048*	
C17	0.37194 (18)	0.5069 (3)	0.32891 (8)	0.0357 (5)	
H17	0.3673	0.5374	0.3651	0.043*	

### Atomic displacement parameters (Å^2^)

**Table:**

	*U*^11^	*U*^22^	*U*^33^	*U*^12^	*U*^13^	*U*^23^
N1	0.0274 (8)	0.0424 (9)	0.0260 (8)	0.0042 (7)	−0.0039 (6)	0.0034 (7)
N2	0.0322 (9)	0.0523 (11)	0.0314 (9)	0.0075 (8)	0.0002 (7)	−0.0007 (8)
N3	0.0316 (9)	0.0526 (11)	0.0330 (9)	0.0102 (8)	−0.0019 (7)	0.0024 (8)
C4	0.0233 (9)	0.0355 (10)	0.0311 (10)	−0.0005 (8)	−0.0026 (7)	0.0002 (8)
C5	0.0341 (10)	0.0432 (11)	0.0315 (10)	−0.0023 (9)	−0.0080 (8)	0.0044 (8)
C6	0.0417 (12)	0.0485 (12)	0.0284 (10)	−0.0087 (10)	−0.0031 (9)	−0.0010 (9)
C7	0.0376 (11)	0.0432 (12)	0.0361 (11)	−0.0059 (9)	0.0075 (9)	−0.0062 (9)
C8	0.0263 (9)	0.0357 (10)	0.0406 (10)	−0.0003 (8)	0.0011 (8)	0.0017 (8)
C9	0.0256 (9)	0.0327 (9)	0.0273 (9)	−0.0029 (8)	−0.0028 (7)	0.0015 (7)
O10	0.0279 (7)	0.0507 (9)	0.0307 (7)	−0.0054 (6)	−0.0097 (5)	0.0121 (6)
C11	0.0307 (10)	0.0430 (11)	0.0383 (11)	−0.0080 (9)	−0.0074 (8)	0.0124 (9)
C12	0.0260 (9)	0.0330 (10)	0.0299 (9)	−0.0056 (8)	−0.0038 (7)	0.0051 (7)
C13	0.0326 (11)	0.0393 (11)	0.0337 (11)	0.0015 (8)	0.0022 (8)	0.0015 (8)
C14	0.0492 (13)	0.0426 (11)	0.0283 (10)	−0.0043 (10)	−0.0074 (9)	0.0020 (8)
C15	0.0340 (11)	0.0382 (11)	0.0473 (13)	−0.0052 (9)	−0.0175 (9)	0.0095 (9)
C16	0.0278 (10)	0.0401 (11)	0.0530 (13)	0.0023 (9)	0.0001 (9)	0.0029 (10)
C17	0.0345 (11)	0.0409 (11)	0.0317 (10)	−0.0044 (9)	0.0018 (8)	0.0012 (8)

### Geometric parameters (Å, º)

**Table:**

N1—N2	1.341 (2)	O10—C11	1.462 (2)
N1—C9	1.357 (2)	C11—C12	1.494 (3)
N1—O10	1.3714 (19)	C11—H11A	0.9900
N2—N3	1.308 (2)	C11—H11B	0.9900
N3—C4	1.374 (3)	C12—C13	1.385 (3)
C4—C9	1.388 (3)	C12—C17	1.393 (3)
C4—C5	1.405 (3)	C13—C14	1.386 (3)
C5—C6	1.370 (3)	C13—H13	0.9500
C5—H5	0.9500	C14—C15	1.380 (3)
C6—C7	1.411 (3)	C14—H14	0.9500
C6—H6	0.9500	C15—C16	1.380 (3)
C7—C8	1.372 (3)	C15—H15	0.9500
C7—H7	0.9500	C16—C17	1.384 (3)
C8—C9	1.396 (3)	C16—H16	0.9500
C8—H8	0.9500	C17—H17	0.9500
			
N2—N1—C9	112.56 (15)	O10—C11—C12	106.54 (15)
N2—N1—O10	120.16 (15)	O10—C11—H11A	110.4
C9—N1—O10	126.85 (16)	C12—C11—H11A	110.4
N3—N2—N1	107.55 (16)	O10—C11—H11B	110.4
N2—N3—C4	108.00 (17)	C12—C11—H11B	110.4
N3—C4—C9	109.55 (17)	H11A—C11—H11B	108.6
N3—C4—C5	130.21 (19)	C13—C12—C17	118.63 (18)
C9—C4—C5	120.21 (18)	C13—C12—C11	120.70 (19)
C6—C5—C4	116.99 (19)	C17—C12—C11	120.66 (18)
C6—C5—H5	121.5	C12—C13—C14	120.7 (2)
C4—C5—H5	121.5	C12—C13—H13	119.7
C5—C6—C7	121.75 (19)	C14—C13—H13	119.7
C5—C6—H6	119.1	C15—C14—C13	120.04 (19)
C7—C6—H6	119.1	C15—C14—H14	120.0
C8—C7—C6	122.2 (2)	C13—C14—H14	120.0
C8—C7—H7	118.9	C16—C15—C14	119.99 (19)
C6—C7—H7	118.9	C16—C15—H15	120.0
C7—C8—C9	115.53 (19)	C14—C15—H15	120.0
C7—C8—H8	122.2	C15—C16—C17	119.9 (2)
C9—C8—H8	122.2	C15—C16—H16	120.0
N1—C9—C4	102.31 (16)	C17—C16—H16	120.0
N1—C9—C8	134.33 (18)	C16—C17—C12	120.70 (19)
C4—C9—C8	123.35 (18)	C16—C17—H17	119.7
N1—O10—C11	109.80 (14)	C12—C17—H17	119.7
			
C9—N1—N2—N3	−1.4 (2)	C5—C4—C9—C8	1.0 (3)
O10—N1—N2—N3	−174.41 (17)	C7—C8—C9—N1	177.9 (2)
N1—N2—N3—C4	1.5 (2)	C7—C8—C9—C4	−0.6 (3)
N2—N3—C4—C9	−1.2 (2)	N2—N1—O10—C11	−74.3 (2)
N2—N3—C4—C5	176.8 (2)	C9—N1—O10—C11	113.8 (2)
N3—C4—C5—C6	−178.2 (2)	N1—O10—C11—C12	−177.11 (16)
C9—C4—C5—C6	−0.3 (3)	O10—C11—C12—C13	−104.8 (2)
C4—C5—C6—C7	−0.7 (3)	O10—C11—C12—C17	76.1 (2)
C5—C6—C7—C8	1.1 (3)	C17—C12—C13—C14	0.3 (3)
C6—C7—C8—C9	−0.4 (3)	C11—C12—C13—C14	−178.85 (19)
N2—N1—C9—C4	0.7 (2)	C12—C13—C14—C15	−0.4 (3)
O10—N1—C9—C4	173.08 (18)	C13—C14—C15—C16	−0.4 (3)
N2—N1—C9—C8	−178.1 (2)	C14—C15—C16—C17	1.2 (3)
O10—N1—C9—C8	−5.7 (4)	C15—C16—C17—C12	−1.2 (3)
N3—C4—C9—N1	0.3 (2)	C13—C12—C17—C16	0.5 (3)
C5—C4—C9—N1	−177.93 (19)	C11—C12—C17—C16	179.64 (19)
N3—C4—C9—C8	179.28 (19)		

### Hydrogen-bond geometry (Å, º)

Cg1 is the centroid of the C12–C17 benzene ring.

**Table:**

*D*—H···*A*	*D*—H	H···*A*	*D*···*A*	*D*—H···*A*
C13—H13···*Cg*1^i^	0.95	2.86	3.685 (2)	145
C16—H16···*Cg*1^ii^	0.95	2.99	3.691 (3)	132

Symmetry codes: (i) −*x*+1, *y*−1/2, −*z*+1/2; (ii) −*x*−1/2, *y*−1/2, *z*.
